# Case report: Cholangiocarcinoma in a choledochal cyst

**DOI:** 10.4103/0971-3026.41836

**Published:** 2008-08

**Authors:** Arti Chaturvedi, JP Singh, Vikas Rastogi

**Affiliations:** Department of Radiodiagnosis, Command Hospital Air Force, Bangalore, India

**Keywords:** Choledochal cyst, cholangiocarcinoma, PET/CT, biliary anomalies

## Abstract

Cholangiocarcinoma is a dreaded complication of unresected choledochal cysts, with an incidence approaching 20-30% in early adulthood. The risk of cholangiocarcinoma remains high where an internal drainage procedure has been performed and the cyst has been partially resected or left unresected. We report a case of cholangiocarcinoma occurring in an unresected choledochal cyst following a drainage procedure in infancy and highlight the role of PET/CT in its diagnosis.

Cholangiocarcinoma is a dreaded complication of unresected choledochal cysts, with an incidence approaching 20-30% in early adulthood.[1] The risk of cholangiocarcinoma remains high where an internal drainage procedure has been performed and the cyst has been partially resected or left unresected.[1,2] We report a case of cholangiocarcinoma occurring in an unresected choledochal cyst following a drainage procedure in infancy and highlight the role of PET/CT in its diagnosis.

## Case History

A 20-year-old unmarried girl reported to the surgical outpatient department with complaints of unrelenting pain in the upper abdomen, vomiting, and loss of weight for 2 months. There was a history of some surgery done for jaundice in early infancy but the details were not available. Clinical examination revealed a jaundiced, cachexic patient, with a rounded mass in the right upper quadrant that was separate from the liver. She was anemic and biochemical tests for liver function revealed a cholestatic type of jaundice.

**Figure 1 (A, B):**
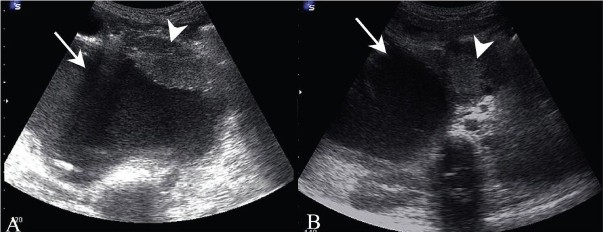
Transverse subcostal USG (A) through the right upper quadrant shows a large cyst (arrow) with eccentric, lobulated soft tissue (arrowhead) along the anterior wall and with debris along the posterior wall. A transverse epigastric scan (B) demonstrates a rounded mass (arrowhead) in the region of the pancreatic head, medial to the large subhepatic cyst (arrow)

**Figure 2:**
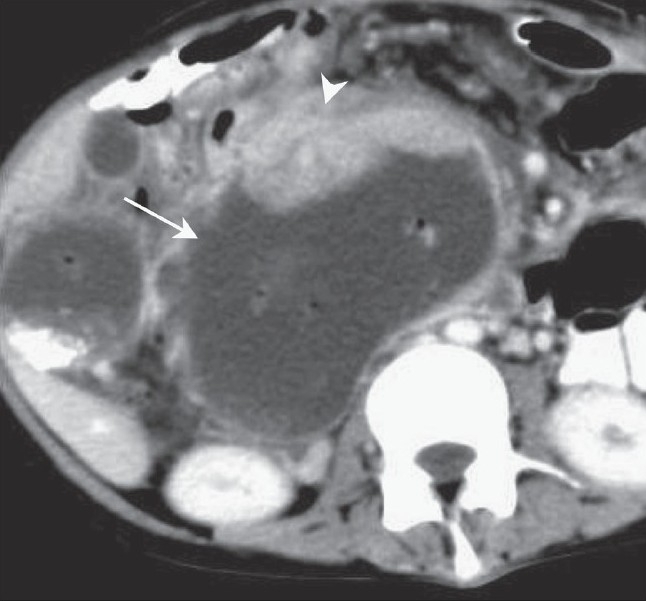
Contrast-enhanced axial CT scan through the inferior part of the cyst shows an enhancing, eccentric, lobulated mass (arrowhead) arising from the anterior wall of the cyst (arrow). Air and oral contrast within the cyst are noted

**Figure 3:**
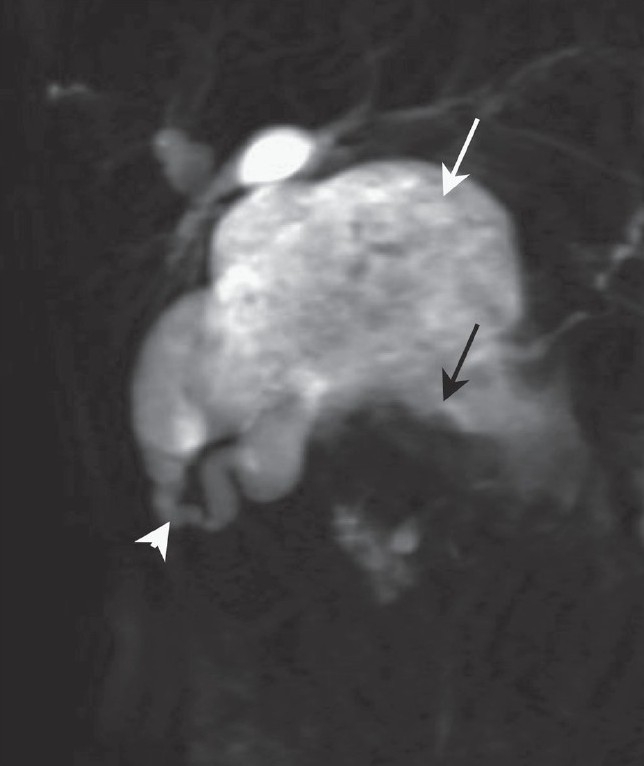
Coronal MRCP image shows the cystic duct (arrow) opening into the right wall of the large cyst (arrowhead); inferiorly, there are multiple, lobulated filling defects (curved arrows) due to the tumor masses

USG revealed a large cystic lesion containing air and debris in the subhepatic region and a lobulated soft tissue mass along the anterior wall of this cyst inferiorly [Figure 1A]. Another separate, 2.5-cm, rounded solid mass was seen in the region of the pancreatic head [Figure 1B]. The common bile duct (CBD) was not separately visualized and there was pneumobilia, with dilatation of the proximal intrahepatic biliary radicles. CT scan revealed a large cystic mass completely replacing the CBD, with reflux of orally ingested iodinated contrast into the cyst as well as the intrahepatic biliary radicles, suggesting a biliary-enteric communication. The intracystic soft tissue mass and the pancreatic head mass showed significant enhancement [Figure 2]. MRCP showed the cystic duct entering the cyst, confirming it to be a dilated CBD [Figure 3]. The intracystic soft tissue showed up as large filling defects along the anteroinferior cyst wall.

**Figure 4:**
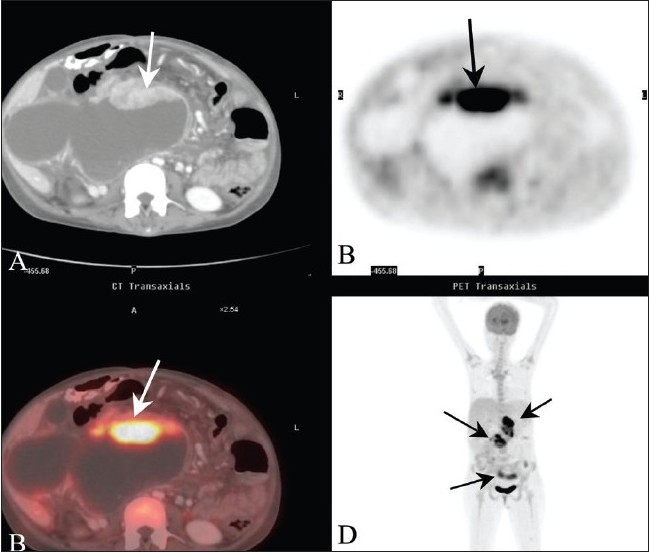
(A-D): Contrast-enhanced CT (A) shows the soft tissue mass (arrow) in the anterior aspect of the cyst, with increased uptake (arrow) in the FDG PET (B) and PET/CT fusion (C) images. The coronal PET image (D) shows additional areas of increased uptake (arrows) in the pancreatic head, pelvis, and left lower quadrant

On the basis of these findings, the diagnosis of a type 1 choledochal cyst, with evidence of a prior cysto-duodenal bypass surgery and the presence of an intracystic cholangiocarcinoma, was made. The patient was unwilling for image-guided fine needle aspiration cytology. A PET/CT performed to further evaluate the intracystic mass showed markedly increased tracer uptake within this soft tissue as well as in the pancreatic head mass (SUV 4.5). Additional peritoneal deposits were also detected in the left lower quadrant and pelvis [Figure 4]. This reaffirmed the suspected diagnosis of cholangiocarcinoma along with intraabdominal spread. The patient was advised chemotherapy; unfortunately, he left the hospital against medical advice and was lost to follow-up.

## Discussion

Choledochal cysts are more prevalent in Asia than in Western countries and approximately 33-50% of reported cases are from Japan.[3] They occur more often in women, with a male to female ratio of 1:3 to 4.[1] Todani's classification of choledochal cysts is at present universally accepted and is as follows:[4]

Type I - Dilatation of hepatic and common bile ducts (40-85%)
Type II - CBD diverticulum (2-4%)
Type III - Intraduodenal CBD dilatation (1.4-5.6%)
Type IV - a Intrahepatic and extrahepatic bile duct dilatation (18-20%)
Type IV - b Multiple extrahepatic cysts (rare)
Type V - Intrahepatic bile duct dilatation (rare)

The classic clinical triad of abdominal pain, jaundice, and abdominal mass is found only in a minority of patients. The clinical presentation largely depends on age - abdominal pain being the most frequent presenting symptom in adults and jaundice in infants.[1] In most patients, USG suffices for the detection and diagnosis of a choledochal cyst. A typical choledochal cyst appears as a water-density mass at the porta or adjacent to the head of the pancreas, with varying degrees of intrahepatic biliary dilatation.[5] A cross-sectional modality such as CT and MRI is usually indicated preoperatively to delineate its relationship with the surrounding structures and also when malignancy is suspected.[1,5] MRCP is increasingly being accepted as a safe, noninvasive substitute for ERCP.[6] It allows direct imaging of the cyst in multiple planes and can demonstrate its communication with the biliary tract. It can also demonstrate any anomaly of the pancreaticobiliary duct junction, which is often considered a possible etiological factor in choledochal cyst formation.[1,6]

Complications of choledochal cysts are the result of stasis and include cholangitis, stone formation, recurrent pancreatitis, cirrhosis, and portal hypertension. Cholangiocarcinoma is a dreaded complication and is attributed to chronic mucosal irritation; it has an incidence of 10-30%. This risk is even higher after drainage procedures.[1,2] Nodular wall thickening and/or an enhancing mass in a choledochal cyst are highly suspicious of malignant change.[5,7] In these high-risk patients, FDG PET/CT may detect early tumors and reveal unsuspected distant metastases and thus impact management.[8]

Because of the high and persistent risk of cholangiocarcinoma, primary excision of extrahepatic choledochal cysts with biliary-enteric anastomosis is the treatment of choice in all cases, even in the absence of symptoms.[1,2] Cholangiocarcinoma developing in a choledochal cyst usually has an adverse outcome because of late diagnosis and a low possibility of resectability.
